# Suppression of creep-regime dynamics in epitaxial ferroelectric BiFeO_3_ films

**DOI:** 10.1038/srep10485

**Published:** 2015-05-27

**Authors:** Y. J. Shin, B. C. Jeon, S. M. Yang, I. Hwang, M. R. Cho, D. Sando, S. R. Lee, J.-G. Yoon, T. W. Noh

**Affiliations:** 1 Center for Correlated Electron Systems, Institute for Basic Science (IBS), Seoul 151-742, Republic of Korea; 2 Department of Physics and Astronomy, Seoul National University (SNU), Seoul 151-742, Republic of Korea; 3 Electronic Materials Research Center, Korea Institute of Science and Technology, Seoul 136-791, Republic of Korea; 4 Department of Physics, University of Suwon, Hawseong, Gyunggi-do 445-743, Republic of Korea

## Abstract

Switching dynamics of ferroelectric materials are governed by the response of domain walls to applied electric field. In epitaxial ferroelectric films, thermally-activated ‘creep’ motion plays a significant role in domain wall dynamics, and accordingly, detailed understanding of the system’s switching properties requires that this creep motion be taken into account. Despite this importance, few studies have investigated creep motion in ferroelectric films under *ac*-driven force. Here, we explore *ac* hysteretic dynamics in epitaxial BiFeO_3_ thin films, through ferroelectric hysteresis measurements, and stroboscopic piezoresponse force microscopy. We reveal that identically-fabricated BiFeO_3_ films on SrRuO_3_ or La_0.67_Sr_0.33_MnO_3_ bottom electrodes exhibit markedly different switching behaviour, with BiFeO_3_/SrRuO_3_ presenting essentially creep-free dynamics. This unprecedented result arises from the distinctive spatial inhomogeneities of the internal fields, these being influenced by the bottom electrode’s surface morphology. Our findings further highlight the importance of controlling interface and defect characteristics, to engineer ferroelectric devices with optimised performance.

Ferroic materials, such as ferromagnets and ferroelectrics, are used in numerous emerging technological applications, including energy harvesting^1^,^2^, actuators^3^,^4^, tuneable microwave devices^5^,^6^, and non-volatile memories^7^,^8^,^9^,^10^,^11^. To optimise the operation speed of such devices, understanding the frequency-dependent response of the active material is crucial. Under *ac* field, ferroic materials typically show hysteretic behaviour that is strongly influenced by changes in the drive frequency 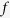. Even though this hysteretic behaviour has been studied for more than 100 years^12^,^13^; the underlying frequency-dependent dynamics remain far from being fully understood.

Hysteresis observed in ferroic materials is due to the motion of domain walls, the boundary between physical regions, *i.e.* domains, with distinct ordering parameters. With disorder, domain wall dynamics can be described by pinning-dominated motion of elastic objects driven by an external force. This concept has become a standard framework for describing a wide variety of physical systems, including vortices in type-II superconductors^14^, dislocation lines in crystals^15^, charge density waves in solids^16^, stripe phases, and domain walls in ferromagnets^17^,^18^,^19^and ferroelectrics^20^,^21^.

Domain wall motion under constant *dc* force can be conceptually separated into three dynamic regimes^21^,^22^,^23^, illustrated schematically in Fig. 1a. At zero temperature, the domain walls are pinned by disorder and do not move under a small driving force, 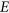. (For ferroelectric domain walls, 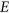 corresponds to external electric field.) Above a critical driving force 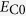, a *depinning* transition occurs. After this transition, the objects have an average velocity *v* that is linearly proportional to 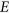; this is the *flow* regime. At finite temperature, thermal effects ‘smear out’ the depinning transition, and a finite velocity is expected for non-zero field. In this case, the movement of the objects in the low field regime is usually described by thermally-activated motion, known as *creep*. Despite significant research on creep-dominated dynamics^16^,^17^,^18^,^19^,^20^,^21^, this regime of domain wall behaviour is not well understood, particularly in the case of *ac* driven field.

In this Article, we explore *ac*-driven hysteretic dynamics of epitaxial thin films of ferroelectric BiFeO_3_ (BFO) grown on SrTiO_3_ (STO) substrates. The BFO layers were deposited in identical growth conditions (as described in Methods), but with different bottom electrodes, La_0.67_Sr_0.33_MnO_3_ (LSMO) and SrRuO_3_ (SRO), and identical top Pt electrodes. Despite the identical preparation conditions, we show here that the *ac*-driven dynamics of the two systems are significantly different. In particular, we find that the SRO bottom electrode gives rise to previously unobserved creep-free hysteretic dynamics, which is the result of a reduced state of disorder. Our findings suggest that judicious choice of bottom electrode can significantly alter domain wall dynamics, and we propose a conceptual framework explaining our observations.

## Results

### Frequency-dependent ferroelectric switching

**Figure 1:**
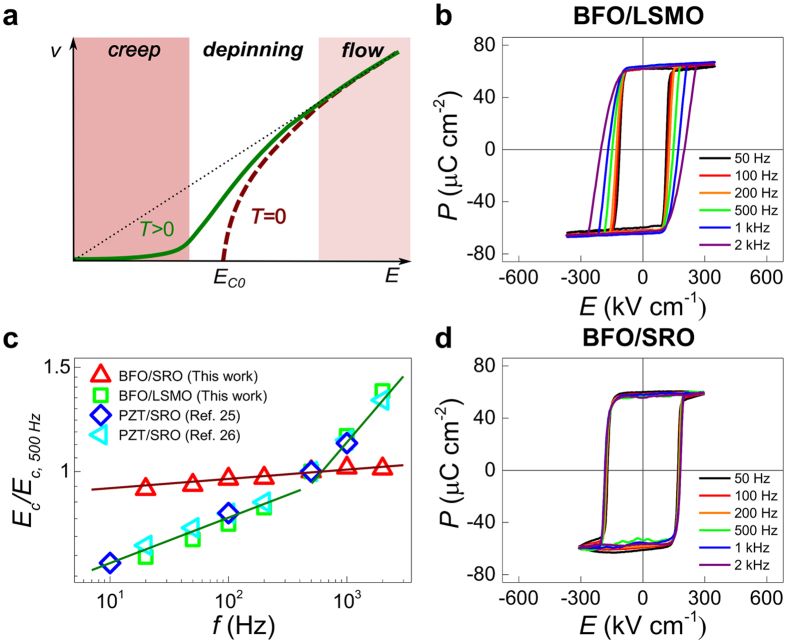
| Scheme of *dc* domain wall dynamics and frequency-dependent hysteretic behavior of BFO films. (**a**) Theoretically-predicted domain wall velocity (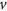) for a pinning dominated system under *dc* electric field. The three dynamic regimes (creep, depinning, flow) are indicated by different colors. 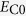 represents a dynamic threshold field at zero temperature. (**b**) Room-temperature polarization-electric field (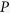
*-*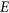) hysteresis loops of BFO/LSMO film. (**c**) log-log 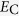-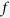 plot of our BFO films with comparison to previously-reported data for PZT films. These data are normalized to the value of 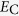 at 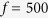 Hz for clarity (see Supplementary Fig. S1 for raw data). The solid lines are fitting results using 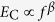. (**d**) Room-temperature 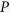
*-*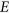 hysteresis loops of BFO/SRO film.

First we consider room-temperature polarisation-electric field (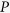-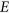) hysteresis loops. 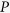-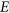 loops measured on Pt/BFO/LSMO capacitors (Fig. 1b) are strongly frequency dependent: higher frequencies induce a decrease in the slope 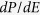, and an increase in the coercive field 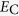. A log-log plot of 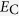 versus frequency (Fig. 1c, green squares) shows that it scales as a power law with 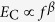 (Refs. 24, 25, 26, 27, 28, 29, 30, 31). These data, when normalised, are consistent with previously-reported data for other epitaxial ferroelectric films^25^,^26^. The two distinct scaling regions observed in this system likely originate from a dynamic crossover between creep and viscous flow regimes^26^,^31^.

In strong contrast, room-temperature 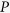-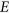 hysteresis loops measured on Pt/BFO/SRO capacitors (Fig. 1d) are virtually frequency independent, and the corresponding plot of 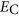-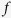 (Fig. 1c, red triangles) shows a single scaling regime with small β. We stress here that these data are fundamentally different from those of typical ferroelectric films, such as epitaxial Pb(Zr_1-*x*_,Ti_*x*_)O_3_ (PZT), whose 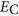-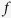 data^25^,^26^ are also shown in Fig. 1c. This suggests that the domain wall motion in our two systems is governed by markedly different *ac*-driven hysteretic dynamics.

### Domain wall propagation

**Figure 2:**
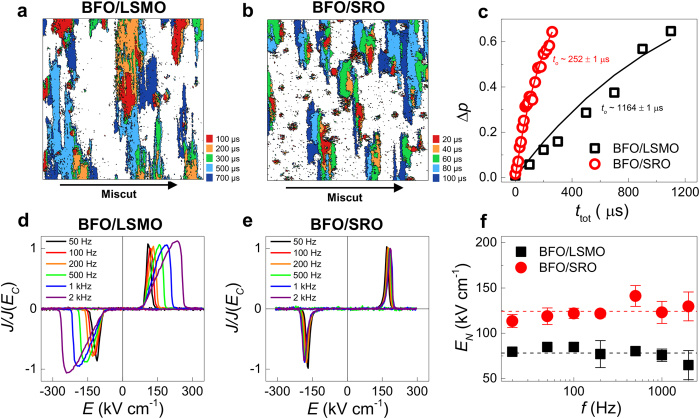
| Domain nucleation and propagation characteristics. Overlaid out-of-plane phase images (25 μm^2^) of switched domains for (**a**) BFO/LSMO and (**b**) BFO/SRO films, measured by stroboscopic PFM. Areas of different colours represent switched domain regions at particular accumulated pulse time 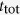. (**c**) Volume fraction of switched polarization (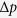) plotted as a function of 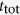. The solid lines show results of the KAI model fitting with 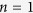. The characteristic switching time 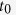 is smaller for BFO/SRO, indicating faster domain wall propagation. Normalized switching current curves of (**d**) BFO/LSMO and (**e**) BFO/SRO. (**f**) Plot of minimum nucleation field (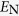) as a function of 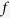. The dashed lines denote the average 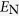 values for each film.

The next step is to examine how the domain walls propagate under applied electric field. We employ stroboscopic piezoresponse force microscopy (PFM)^32^,^33^,^34^, a versatile technique that allows direct visualisation of time-dependent polarisation changes (see Methods). Overlaid out-of-plane PFM phase images for the BFO/LSMO and BFO/SRO films are presented in Fig. 2a,b where the colours indicate the domain configuration after the application of switching pulses of a particular accumulated time 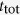. Comparison of these images, keeping in mind the different time increments in each case, shows that for BFO/SRO, after a shorter accumulated time, a larger area is switched. This implies a higher domain wall velocity in BFO/SRO.

Quantitative information on the domain wall dynamics is obtained using the Kolmogorov-Avrami-Ishibashi (KAI) model^35^:


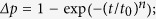


where 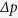 is the volume fraction of switched polarisation, 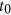 the characteristic switching time, and 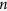 the geometric dimension of the domains. The experimental 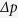 for our BFO films, estimated from analysis of the PFM images, is plotted against 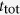 in Fig. 2c. A monotonic increase in Δ*p* is observed, and its growth is much more rapid for the BFO/SRO film. Fitting these data to the KAI model (solid lines in Fig. 2c) with 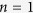, consistent with the observed 1D-like domain propagation (see the Supplementary Information for details), yields 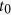 values for BFO/LSMO and BFO/SRO of 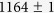 μs and 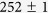 μs, respectively. Since domain wall velocity 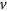 is inversely proportional to 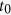 (Ref. 35), we conclude that the domain walls in BFO/SRO propagate about five times faster than in BFO/LSMO.

### Domain nucleation

More careful inspection of the overlaid PFM images reveals details of the nucleation process. For the BFO/LSMO film (Fig. 2a), domain nuclei of opposite polarisation appear over a wide 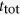 range, and, more specifically, nuclei start to appear at 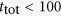 μs but newly-nucleated domains continue to appear even at 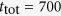 μs. In epitaxial PZT films, Kim *et al.* showed that the number of nuclei is linearly proportional to 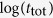, suggesting a broad distribution of activation energies for nucleation^33^. This picture is consistent with our present observation in the BFO/LSMO system.

For the BFO/SRO film (Fig. 2b), on the other hand, the nucleation of all domains takes place within 20 μs, and subsequent domain switching systematically occurs in regions adjacent to already-switched domains. The new formation of domains does not occur after the initial surge of nucleation at ~20 μs; that is, all further domain switching takes place via sideways growth. This suggests that the nucleation process for the BFO/SRO system can be defined by a narrower distribution of activation energies.

A quantitative comparison of the nucleation energies in the two systems is obtained through analysis of the time-dependent switching current density (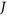) during polarisation reversal (see Methods). 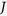-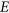 hysteresis curves (in which 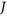 is normalised to its value at the coercive field) for both samples at various frequencies are presented in Fig 2d,e. For BFO/LSMO, increasing frequency causes the 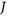-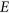 curves to broaden significantly, consistent with i) the nucleation process taking place over a rather wide 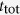 range, and ii) lower domain wall velocity. In contrast, for BFO/SRO, the 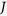-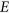 curves remain very sharp and show negligible frequency dependence (Fig. 2e), coherent with the reasoning that nucleation occurs in a narrow time window and that switching is completed more quickly via faster domain wall propagation. From the 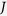-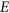 curves, we extracted the *minimum* electric field value (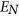) at which domains start to nucleate, taking it to be the value of 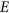 when 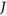 reaches 1% of 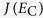. The 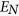 values for BFO/LSMO and BFO/SRO (Fig. 2f, solid black squares and red circles, respectively) indicate that the first few nuclei of opposite polarisation appear at around the same value of field, regardless of frequency. Importantly, the 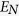 value for BFO/LSMO is smaller by a factor of about 1.6 than that for BFO/SRO, suggesting, perhaps surprisingly, that the nucleation process commences at *lower* 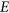 in BFO/LSMO.

### Surface morphology

**Figure 3:**
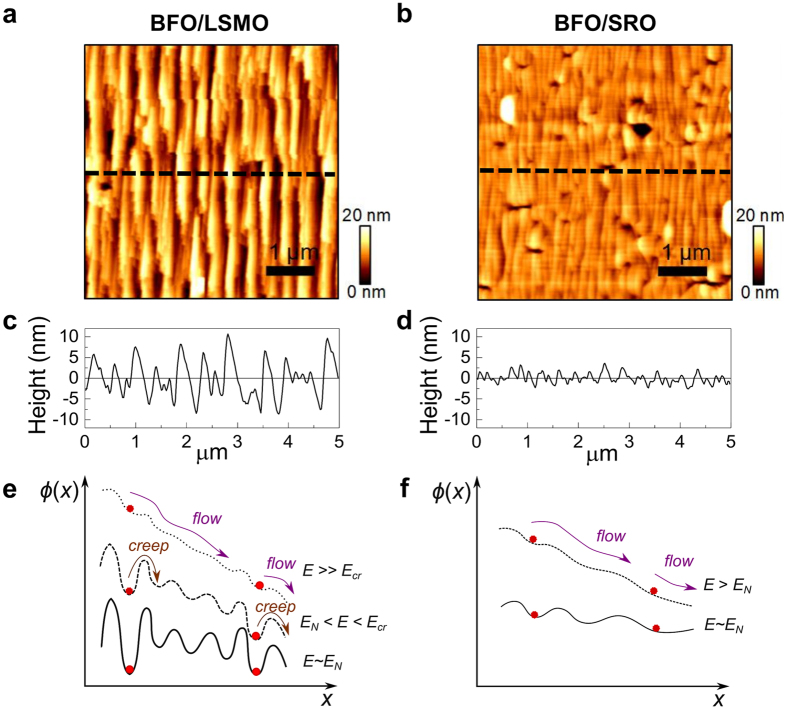
| Surface morphology and schematics of the energy landscape in BFO thin films. AFM topography images of (**a**) BFO/LSMO and (**b**) BFO/SRO films. Line profiles of (**c**) BFO/LSMO and (**d**) BFO/SRO surfaces, taken along the dashed lines in (**a**) and (**b**). Clear step-bunching is observed for both BFO films. Schematic diagram of pinning energy landscape (φ(*x*)) in (**e**) BFO/LSMO and (**f**) BFO/SRO films. The red circles denote *positions* at which nucleation occurs. The arrows represent domain wall motion, depending on applied field 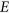 and the pinning energy barrier.

Since, in ferroelectric thin films, nucleation occurs predominantly at the surface and/or interface^33^,^36^,^37^ of the film, the surface topography of the BFO layer for the two cases can give us hints as to why the nucleation processes differ. Atomic force microscopy (AFM) topography images (Fig. 3a,b) reveal that they have markedly different surface morphologies: BFO/SRO, with root-mean-square (*rms*) roughness ~1.50 nm, has a much flatter surface than BFO/LSMO (*rms* roughness ~3.86 nm). The distinction is seen more clearly in corresponding line scans (Fig. 3c,d) which indicate that the height variation of the BFO/SRO film is considerably smaller than that for the BFO/LMSO film. Since domain wall pinning is more likely to occur for the film with larger surface roughness^38^,^39^, these topography images suggest that the BFO/LSMO film has a *higher density* of *stronger* pinning sites.

### Temperature-dependent hysteretic dynamics

**Figure 4:**
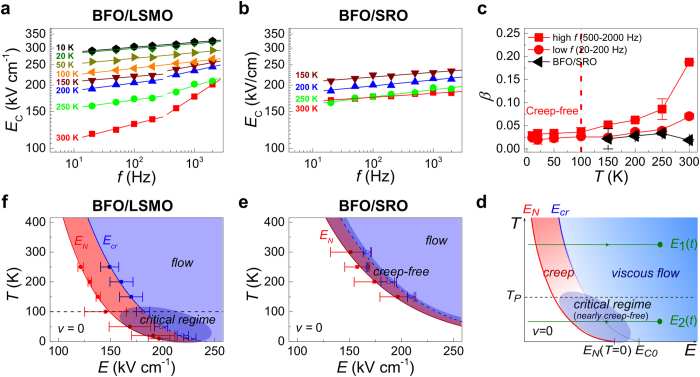
| Temperature (*T*)-dependent hysteretic dynamics. log-log 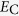-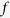 plot for (**a**) BFO/LSMO and (**b**) BFO/SRO film at various temperatures. (**c**) Plot of β values with respect to 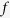. The β values of BFO/LSMO at low 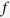 (20-200 Hz) and high 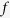 (500-2000 Hz) are represented by red circles and red squares respectively. The left-side of the (red) dashed line indicates the low-temperature creep-free regime of BFO/LSMO. The β values for BFO/SRO are represented as black triangles. (**d**) Phase diagram of hysteretic domain wall dynamics under *ac*-driven field. The solid red lines 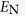 and blue 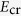 show the dynamic crossover between no nuclei, creep, and viscous flow regimes. The regimes where creep or viscous flow motion govern domain wall dynamics are shaded red and blue, respectively. The green lines 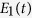 and 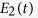 represent the first 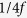 of applied *ac* field at two different temperatures. The horizontal dashed line denotes the temperature 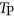, related to the typical pinning energy 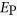. Phase diagrams derived from (**f**) BFO/LSMO data and (**e**) BFO/SRO data. Red circles indicate 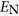 and blue solid squares and open squares present 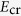 and the critical regime, respectively. Lines are guides to the eye.

To further understand the effects of domain wall pinning, we now turn to the influence of temperature on the switching dynamics. We measured 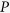
*-*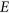 hysteresis loops for the two systems over a wide temperature range. Decreasing the temperature to below 100 K suppresses the two-scaling behaviour exhibited by the BFO/LSMO film, as evidenced by 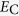-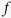 curves (Fig. 4a). The *β* values at high (500–2000 Hz) and low (20–200 Hz) frequency scaling regimes (Fig. 4c, red squares and circles respectively) merge, and the corresponding *β* value is rather small. Since the two-scaling behaviour is a hallmark for the existence of the dynamic crossover between creep and viscous flow regimes^26^,^31^, the merging of the exponent β at low temperature signifies an essential change in domain wall dynamics; namely, the suppression of the creep regime.

The 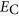-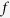 plot for the BFO/SRO film (Fig. 4b), derived from hysteresis measurements carried out at various temperatures, does not exhibit a change in scaling behaviour. Rather, *β* is invariant down to low temperature, indicating that the BFO/SRO system does not exhibit a dynamic crossover, even at room temperature. Importantly, at low temperature the 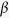 values for the BFO/LSMO film are comparable to those for the BFO/SRO film, implying that the dynamics of the two systems are similar in this temperature window.

### Discussion

For ferromagnetic materials, Nattermann *et al.* developed a theory on *ac* hysteretic domain wall propagation dynamics^40^. The theory considers that a domain wall exists for any external field, and it predicts a *relaxation* regime of 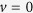 for values of applied field lower than that needed to enter the creep regime. In this relaxation regime, a domain wall cannot overcome the depinning energy from a pinning site before the external *ac* field changes its polarity. Using this general framework of pinning-dominated dynamics in a ferroic system, we have extended this theory to explain our present observations.

In Fig. 4d, we present the proposed schematic phase diagram for *ac*-field hysteretic dynamics of domain wall formation and propagation in an epitaxial ferroelectric film. (A detailed explanation of its construction can be found in the Supplementary Information). Here the main difference between this phase diagram and that of Ref. 40 is that the relaxation regime of Natterman’s theory is replaced by a regime labelled ‘no nuclei,’ for electric field values lower than that required for nucleation; that is 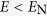. Here there is no domain wall in the system, and therefore, by definition 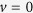.

It is important to emphasise the temperature 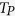 which defines the upper boundary of the ‘critical regime.’ Above this temperature, the thermal energy is high enough to allow hopping and as a result the ‘creep’ regime separates the ‘no nuclei’ and ‘viscous flow’ regimes. The value of field required to transition from creep to flow dynamics is the dynamic crossover field 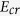. For temperatures below 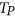, the ‘critical regime’ corresponds to behaviour such that the thermal energy is not sufficient to allow creep motion, and therefore upon increasing field, domain wall dynamics pass directly from ‘no nucleation’ to ‘flow.’ In this region creep motion is suppressed, and since the dynamic crossover does not exist, 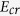 cannot be defined.

Using this phase diagram for *ac* hysteretic dynamics, we explain the temperature-dependent 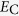 values exhibited by the BFO/LSMO system (Fig. 4a). During *ac* hysteresis loop measurement, the amplitude of the electric field is varied periodically between 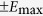, where 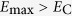. During the first quarter of one period, *i.e.* 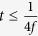, 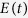 increases from zero to 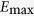.

For 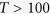 K, the *ac* measurement can be considered as following the path defined by 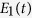, passing through the dynamic crossover at 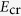. At low frequency, polarisation reversal is completed well before the flow regime is reached. At high frequency, however, 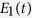 passes through the creep regime so quickly that switching is not completed until after the flow regime has been entered. This interpretation is consistent with the different high- and low-frequency β values in Fig. 4a.

In contrast, for 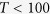 K, the *ac* measurement can be described by the path 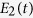. Since this path does not pass through the crossover 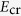, domain walls propagate, regardless of the drive frequency, without experiencing creep motion. As a consequence of this suppression of the creep regime, the distinct high- and low-frequency β values merge (Fig. 4c). The low-temperature value of β, *i.e.* 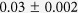, is the result of creep-free domain dynamics in the critical regime. Since, at present, the understanding of β is based solely on phenomenological studies, more theoretical research on *ac* hysteresis dynamics is important to shed further light on these processes.

To strengthen our predictions, we constructed phase diagrams describing the hysteretic dynamics of our BFO films (Fig. 4e,f) from the experimental data, based on the analysis described in Ref. 26 (see Methods). In these figures, red symbols denote experimentally-determined values of 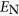, while blue symbols represent values of 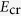. *Solid* blue symbols correspond to 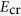 values in the temperature window where the dynamic crossover exists (*i.e.* the behaviour involves the creep regime), while *open* blue symbols denote hypothetical 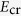 values in the temperature window where the dynamic crossover does not exist; that is, the creep-free regime. For BFO/LSMO (Fig. 4f), the creep, flow, and critical regimes are all observed in the phase diagram, consistent with the aforementioned hypotheses. For the BFO/SRO system (Fig. 4e), however, the values of hypothetical 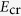 are comparable (within experimental uncertainty) to the values of 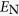, implying that the creep regime is supressed, for all considered temperatures. It is important to remember that the mechanism for creep suppression in the two systems is different: for BFO/LSMO at low temperature it is due to the elimination of thermally-activated hopping, while for BFO/SRO it is related to the reduced state of disorder.

With this detailed understanding of the domain dynamics of the two systems, we now provide an intuitive explanation, at the microscopic level, for our observation of nearly creep-free behaviour. The variation in morphology observed for the SRO and LSMO bottom electrodes (Supplementary Information Fig. S5) is much less pronounced than the BFO surface morphology differences (Fig. 3a,b). This observation can be understood in terms of the growth process. Typically, the growth of SRO occurs in the step-flow mode^41^, yielding a uniform and atomically-flat SRO surface. LSMO, on the other hand, typically grows in a layer-by-layer mode^42^ whereby the atoms are deposited at random positions on the surface. Despite the relatively small difference in surface morphology of the different bottom electrodes, for LSMO the widely-distributed atoms may act as seeds for the subsequent BFO growth, giving rise to an increased dislocation density and surface roughness (Fig. 3a) of the resulting BFO layer. In ferroelectric thin films, nucleation usually occurs either at the interface or at the surface^33^,^36^,^37^; therefore a rougher top surface causes domain nucleation to occur at lower values of applied field, and the corresponding nucleation activation energies will be described by a wider distribution.

The surface roughness may also strongly affect the domain pinning dynamics in our BFO films. A possible origin of domain wall pinning in ferroelectric films is the locally-induced internal electric field. When the BFO film is grown, a structural change (*e.g*. strain relaxation) occurs along the step-terrace edge, generating a locally-induced internal field^43^, which can be described by the pinning potential energy landscape 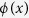. Domain wall pinning sites, located at local minima of 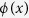, hinder domain wall motion. Due to the rougher surface morphology, 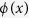 for BFO/LSMO has a much larger variation and many pinning sites with large energy barriers (solid line in Fig. 3e). Just after its nucleation, a domain wall is held by a pinning site and its motion is described by thermally-activated creep (dashed line in Fig. 3e). At higher applied field, flow motion appears (dotted line in Fig. 3e). In contrast, for the BFO/SRO film, 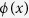 has fewer pinning sites with a smaller variation in their strength (solid line in Fig. 3f). Consequently, the domain wall experiences flow motion immediately after nucleation, allowing it to have a high propagation velocity (dashed line in Fig. 3f).

Our finding of an almost creep-free regime at room temperature is rather surprising, since most existing reports on epitaxial ferroelectric domain wall dynamics demonstrate the existence of this regime. Our work clearly shows that by carefully controlling the state of disorder, in this case through the properties of the bottom electrode, we can engineer ferroelectric devices with substantially improved performance. These results are general and this technique can easily be extended to other systems that are governed by the same physical laws, *e.g*. ferromagnetic systems. In addition to fundamental interest of ferroelectric and ferromagnetic switching dynamics, our results present a clear approach for improving device architectures where switching speed is crucial and frequency dependence of the switching process must be avoided; for instance in high-speed actuators or memory devices.

In summary, we have presented a strategy for obtaining creep-free *ac* hysteresis dynamics in epitaxial ferroelectric thin film devices. Through detailed temperature-dependent hysteresis and scanning-probe microscopy-based ferroelectric switching measurements, we revealed that BFO films grown on SRO bottom electrodes exhibit switching dynamics that are markedly different from those displayed by BFO grown on LSMO bottom electrodes. In particular, in the BFO/SRO system, the typically-observed frequency dependence of 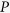
*-*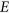 hysteresis is suppressed, and we find that domain wall propagation velocity is much higher. We attribute this intriguing behaviour to the markedly different morphology of the SRO and LSMO layers, which in turn influences the internal fields and energy landscape in the ferroelectric BFO layer. This work is a clear demonstration that domain dynamics in ferroic systems can be controlled by tuning the nature of disorder.

## Methods

### Thin film fabrication

Epitaxial BiFeO_3_ (BFO) thin films were deposited by pulsed laser deposition using a KrF excimer laser (λ = 248 nm) on bottom electrodes of epitaxial La_0.67_Sr_0.33_MnO_3_ (LSMO) or SrRuO_3_ (SRO), prepared on STO substrates with a 4° miscut toward the [100] direction. The BFO thickness, measured by scanning electron microscopy (SEM) on the sample edge, was 280 nm on LSMO and 232 nm on SRO. Further details on the structural and physical properties are published in previous reports^43^,^44^. Capacitor structures 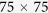 – 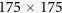 μm^2^ in size were fabricated by depositing 40 nm thick Pt by *dc* magnetron sputtering and patterning using conventional photolithography.

### Electrical measurements


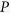
*-*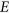 hysteresis loops and switching currents were measured using a AixACCT TF Analyzer 2000, employing a triangular electric field waveform over the frequency range 50–2000 Hz. 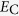 was extracted from the loops according to average of the values of 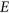 where 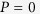 obtained at positive and negative fields. An imprint of 5–15 kV/cm was observed. This imprint has been removed in Fig. 1b,d so as to clearly compare the frequency-dependent characteristics of the two systems. Since the imprint is considerably smaller than 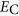, this small correction does not impact the experimental findings or outcome of this study. Temperature dependent 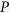
*-*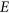 hysteresis loops were measured using a low-temperature cryostat using liquid helium.

### Stroboscopic piezoresponse force microscopy

(PFM) was performed using a Park Systems XE-100 scanning probe microscope with commercially-available non-conductive silicon tips (NSC36/AIBS, Mikromasch); images were obtained by applying a harmonically oscillating electric field (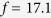 kHz) using a separate needle probe in contact with the top electrode^32^,^33^,^34^. The converse piezoelectric response was detected using a lock-in amplifier (SR830, Stanford Research Systems), yielding amplitude and phase signals. For domain growth measurements, square pulses with amplitude given by 1.1 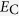 (at 2 kHz) from a function generator (FG300 Yokohama) were applied to the sample’s lower electrode while the tip was grounded. The pulse width was 100 μs for BFO/LSMO and 20 μs for BFO/SRO, so chosen to be adapted to the different domain wall velocities of the two systems. Before PFM measurements, the BFO film was fully poled in one direction, then the opposite voltage was applied as a series of rectangular pulses with constant amplitude. Between the pulses, PFM imaging was performed (Supplementary Fig. S2). The total applied time 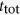 is defined as the sum of the time during which 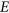 is applied. We confirmed the validity of our stroboscopic PFM measurements using variable pulse widths, following the procedure described in Ref. 34. We found that the switched domain state was preserved before and after PFM image acquisition without evidence of polarisation relaxation or back-switching, confirming that the obtained PFM images are true representations of the domain wall configuration after applying a constant electric field of duration 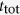.

### Construction of experimental phase diagram

Fig. 4e,f was constructed using the following procedure. At room temperature, we define 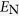 as the 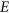 value where the switching current reaches 1% of 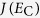. At low temperature, due to the higher noise level of the switching current, we instead used the 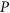
*-*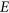 hysteresis loops (Supplementary Fig S4), defining 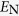 as the crossing point of the average lines of 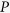
*-*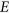 hysteresis before and after switching. The 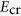 values were taken as the 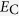 value at the frequency at which dynamic crossover occurs; that is, the frequency at which the slope of the 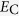-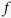 graph changes.

## Additional Information

**How to cite this article**: Shin, Y. J. *et al.* Suppression of creep-regime dynamics in epitaxial ferroelectric BiFeO_3_ films. *Sci. Rep.* **5**, 10485; doi: 10.1038/srep10485 (2015).

## Supplementary Material

Supplementary Information
