# Supplementary material for: Suppression of creep-regime dynamics in epitaxial ferroelectric BiFeO3 films
Source: Sci Rep. 2015 May 27;5:10485. doi: 10.1038/srep10485 (PMC4444839; doi:10.1038/srep10485)
Supplement: Supplementary Information [file srep10485-s1.pdf]

Supplementary Information

Suppression of creep-regime dynamics in epitaxial ferroelectric $BiFeO_3$ films

Y. J. Shin[1,2,*], B. C. Jeon[1,2,*], S. M. Yang[1,2], I. Hwang[3], M. R. Cho[2], D. Sando[1,2], S. R. Lee[1,2], J.-G. Yoon[4], and T. W. Noh[1,2★]

[1]Center for Correlated Electron Systems, Institute for Basic Science (IBS), Seoul 151-747, Republic of Korea

[2]Department of Physics and Astronomy, Seoul National University, Seoul 151-747, Republic of Korea

[3]Electronic Materials Research Center, Korea Institute of Science and Technology, Seoul 136-791, Republic of Korea

[4]Department of Physics, University of Suwon, Hawseong, Gyunggi-do 445-743, Republic of Korea

*These authors contributed equally to this work

★e-mail: twnoh@snu.ac.kr

I. Raw data of Fig. 1c

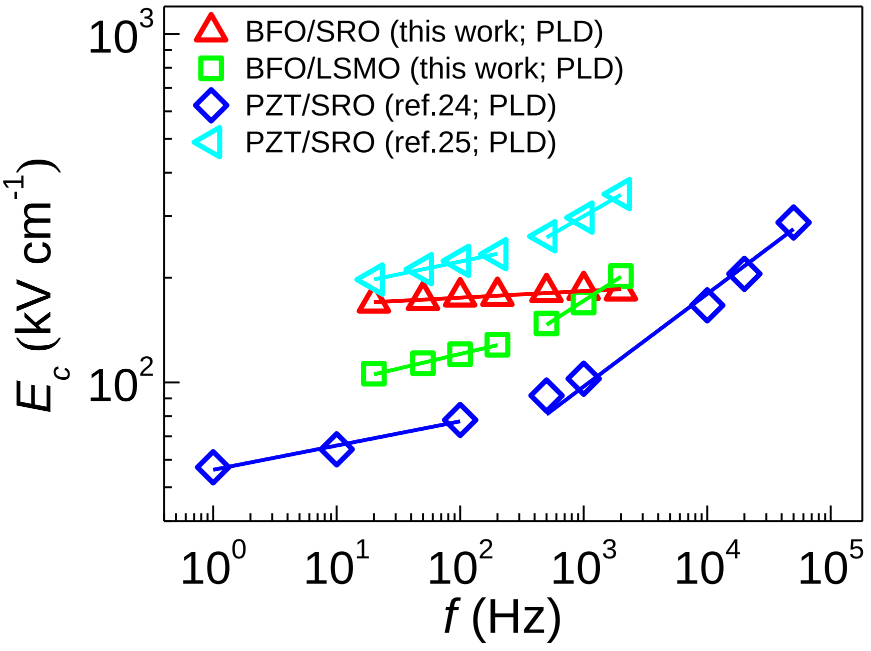

Supplementary Figure S1 | log(E_C)-log(f) plot of BFO and PZT films Raw data of Fig. 1c (*i.e.* without normalization). The solid lines represent fitting results using $E_C \propto f^{\beta}$. A clear difference in the f-dependent E_C values is seen in this figure.

II. Domain wall nucleation and propagation

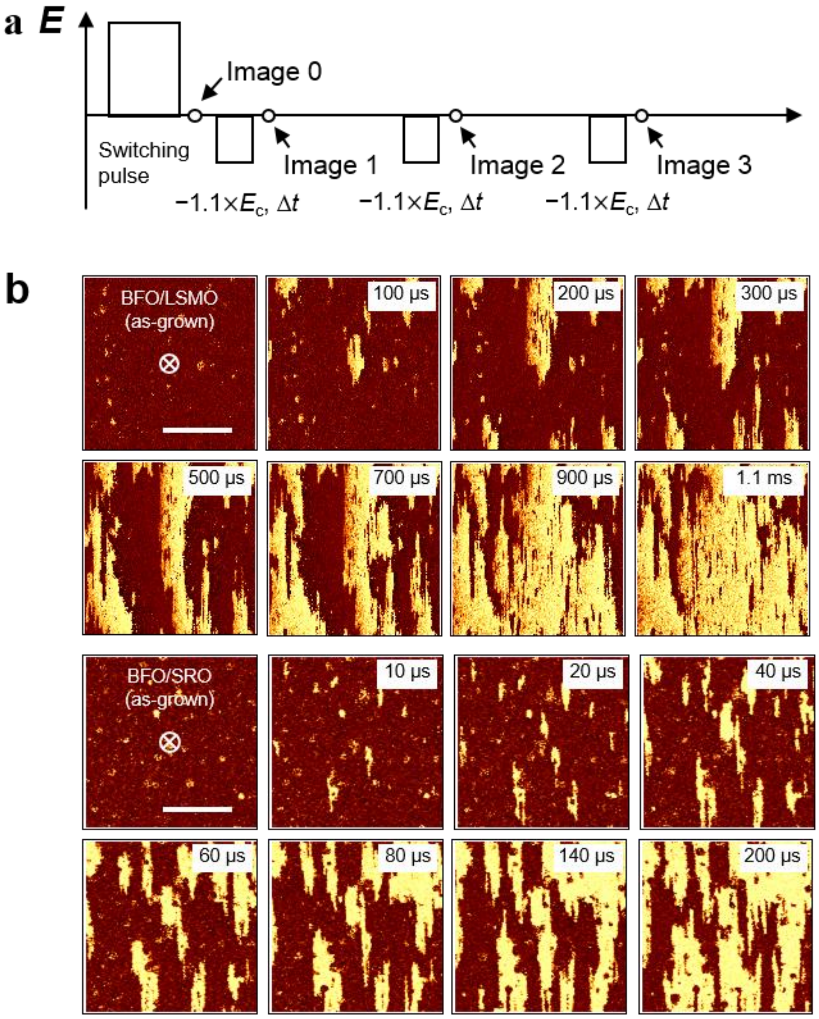

Supplementary Figure S2 | Stroboscopic PFM measurement (**a**) Schematic of applied E pulses. Before measurement, a strong switching pulse is applied for ~1 ms to create a mono-domain state. The sequence of E pulses are applied in a certain time interval Δt. The amplitude of the pulses is chosen to be $1.1E_C$ at 2000 Hz. The Δt are 100 μs and 20 μs for BFO/LSMO and BFO/SRO, respectively. Between each E pulse, PFM image scans are performed. (**b**) The obtained PFM images by the sequence described in (**a**). Bright and dark regions represent domains with upward and downward polarization, respectively. Scale bars, 10 μm. All PFM phase images were obtained at the same location of each film.

The salient feature of these images is that the domains exhibit a highly elongated shape, and propagate preferentially in the vertical direction. Since the substrate miscut is along the horizontal (arrows in Fig. 2a,b), we infer that the domain walls move much faster *along* the step edge than in a direction parallel with the miscut, giving rise to anisotropic quasi-one dimensional (1D) propagation[1].

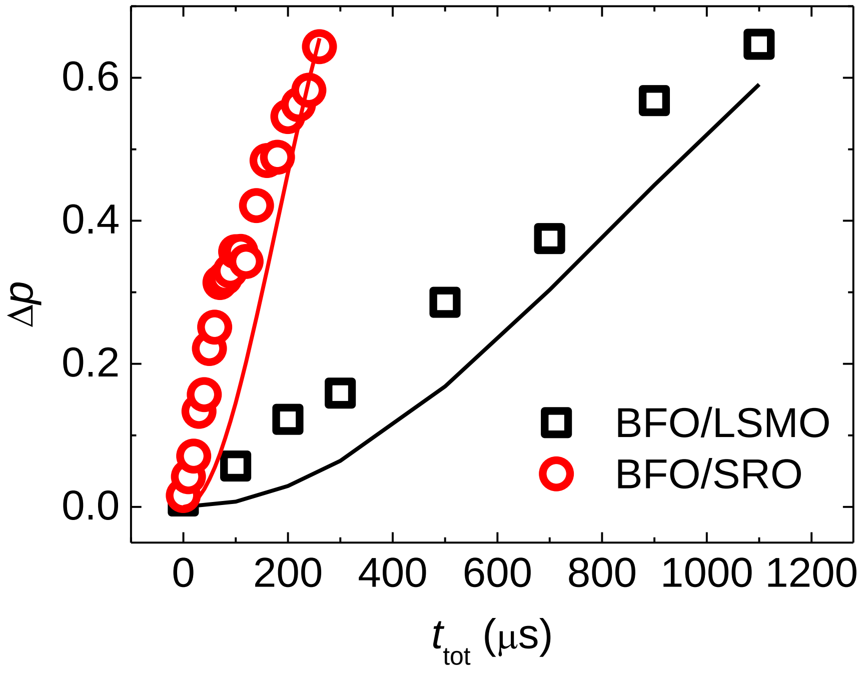

Supplementary Figure S3 | KAI model fitting with $n = 2$ The same data as Fig. 2c, with KAI model fitting results using $n = 2$. In contrast to the results with $n = 1$ in Fig. 2c, the fitting results do not accurately describe the data. This indicates that the domain wall motion in the BFO films is closer to 1D-like motion than typical 2D motion.

III. Discussion of hysteretic phase diagram of *ac*-dynamics

For fully-saturated P-E hysteresis loop measurements, all domains are switched to one mono-domain at the maximum value of field. For a reversed domain to nucleate, it must first overcome the activation energy barrier. Since, in epitaxial films, the activation barrier is generally higher than the depinning barrier[2], the dynamics of a freshly-nucleated domain are described by the creep regime. For this reason, we replace the relaxation regime of Natterman's theory by a regime labelled '*no nuclei*'. In this case, the existence of the *v*=0 regime below $E_{\rm N}$ is due to the fact that there is no domain wall in the system.

We now consider the typical pinning energy of a domain wall[36], $E_{\rm P}$, and we define a temperature $T_{\rm P} = E_{\rm P}/k_{\rm B}$, which can be thought of as a 'pinning temperature'. For temperatures above this threshold, *i.e.* $T > T_{\rm P}$, thermally-induced hopping (creep) is possible and the hysteretic behaviour of the system exhibits a dynamic crossover at $E_{\rm cr}$, separating the creep and viscous flow regimes. In this temperature window, a domain wall first undergoes motion in the creep regime, after which, upon increasing E, it can enter the flow regime. It is important to remember that this boundary is not an abrupt transition but a crossover (as in Fig. 1a), whereby a gradual change occurs over a rather broad E range around the transition threshold.

For $T \ll T_{\rm P}$, that is, when the thermal energy is lower than the typical pinning energy, the critical depinning regime plays a role in the hysteretic dynamics. In this temperature window, the domain propagation is strongly pinned by disorder until the drive field reaches a dynamic threshold E_{C0}. At $E \approx E_{\rm C0}$, the domain undergoes a pinning-depinning transition and begins to propagate (Fig. 1a, red dashed line) with velocity $v \propto (E - E_{C0})^{\theta}$, where the critical exponent θ is determined by pinning forces and the fractal nature of the system. Since, in this case, v is not governed by thermally-activated hopping, creep motion is suppressed, and $E_{\rm cr}$ has no meaning. In other words, immediately after a given domain wall nucleates, it passes directly into the flow regime without experiencing creep motion.

As described in Fig 4d, an *ac* measurement is performed along the horizontal lines (*e.g.* $E_1(t)$). The measurement frequency defines how quickly the *ac* field moves on this line. Depending on frequency, the absolute time the field stays in each regime is different (see Supplementary Fig 5c), explaining the origin of the different β values in the f-E_C plot. The frequency value where β changes indicates that half of the polarisation is switched at the boundary of creep and flow regimes. Therefore, at the crossover frequency, $E_C = E_{cr}$.

IV. Construction of experimental hysteretic phase diagram

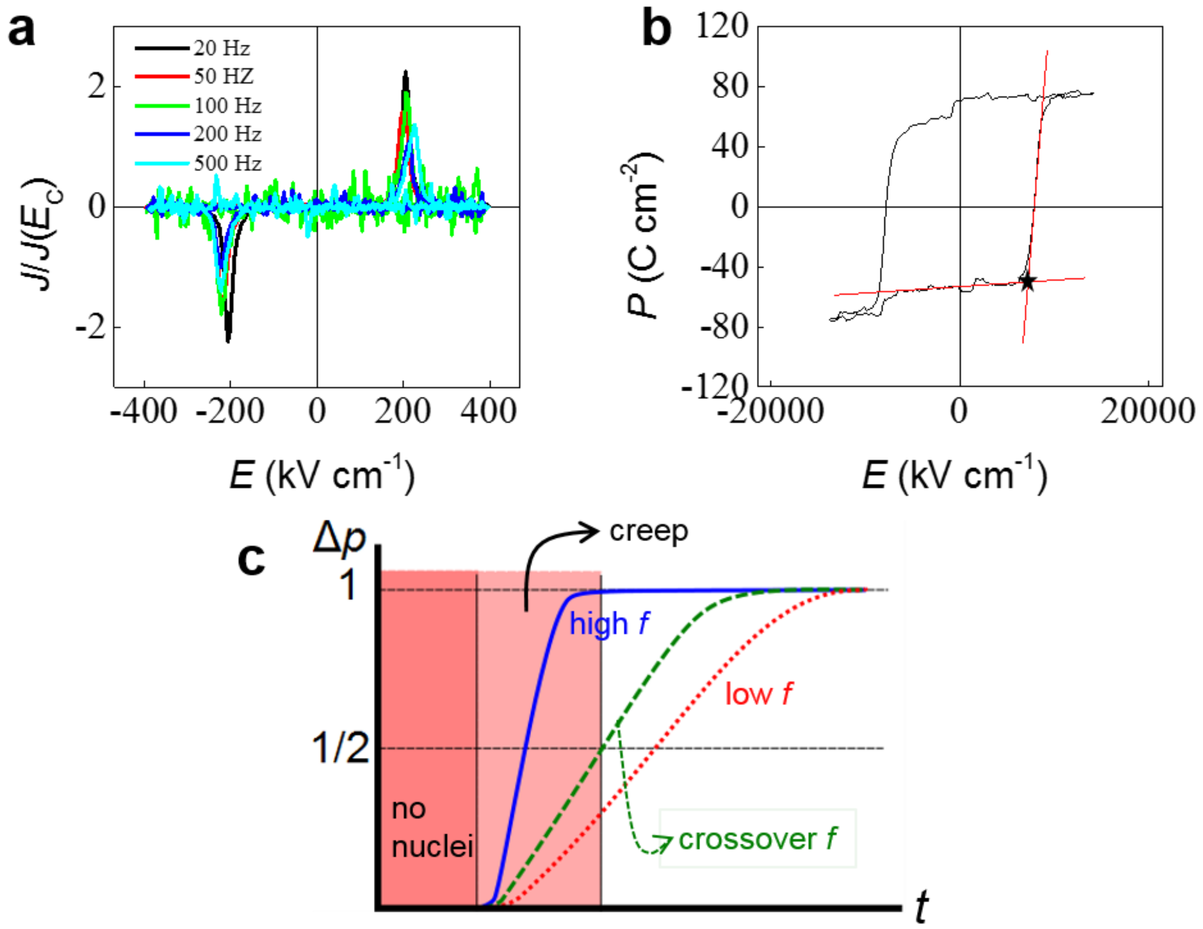

Supplementary Figure S4 | Constructing the phase diagram **(a)** The switching current for BFO/LSMO at 20 K. **(b)** *P-E* hysteresis loop of BFO/LSMO at 20 K using 500 Hz of *ac* field. **(c)** The schematic describing how f can give different β vales in log(f)-log(E_C) plot.

We point out here that the absence of T_P for BFO/SRO is not the result of the limited temperature range of the measurements. In fact, the mechanism for the creep-free regime in BFO/LSMO at low temperature is fundamentally different from the mechanism for creep-free behaviour in BFO/SRO at higher temperatures. To understand this difference, consider $T_P = E_P/k_B$, (where k_B is Boltzmann's constant) the pinning energy barrier required (>300 K) for the system to exhibit the critical region, is not consistent with the fast domain wall propagation observed in the BFO/SRO system. Note that the E_N value is quite large for this film (Fig. 2f). So when a reversed domain becomes nucleated, the domain walls start to flow without creep motion.

V. Another possible origin of surface morphology effect on domain wall pinning dynamics

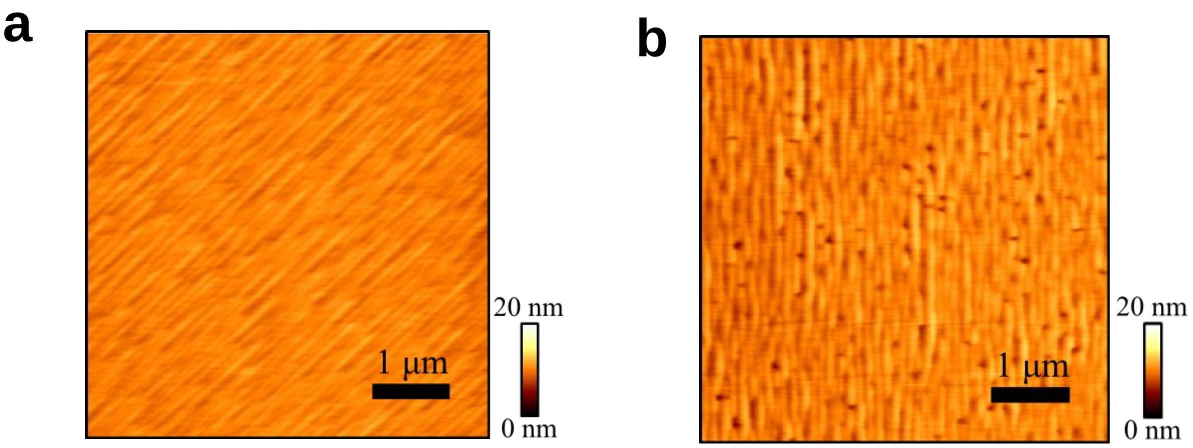

Supplementary Figure S5 | Surface morphology of SRO and LSMO The surface morphologies of **(a)** LSMO and **(b)** SRO The RMS roughness of (**a**) and (**b**) are 0.571 nm, 1.014 nm, respectively

Ahluwalia et al. claim that surface morphology can significantly affect polarization switching of ferroelectrics[3]. According to this theory, surface heterogeneities produce inhomogeneities in the internal E field in the film. This inhomogeneous field produces preferential sites for nucleation. These sites also hinder subsequent domain wall propagation. Note that the inhomogeneous E act the same role as quenched defect of FE films for both nucleation[4–6] and propagation[7–11] of domain walls.

Supplementary references

1. Kim, T. H. *et al.* Electric-field-controlled directional motion of ferroelectric domain walls in multiferroic $BiFeO_3$ films. *Appl. Phys. Lett.* **95,** 262902 (2009).

2. S. M Yang H. N. Lee, T. K. Song and Jong-Gul Yoon, J. W. H. Quantitative analysis of nucleation and growth of ferroelectric domain in epitaxial $Pb(Zr,Ti)O_3$ thin films. *J. Korean Phys. Soc.* **55,** 820–824 (2009).

3. Rajeev, A., Nathaniel, N. & David, J. S. Surface morphology effects on polarization switching in nanoscale ferroelectrics. *Nanotechnology* **20,** 445709 (2009).

4. Kim, D. J. *et al.* Observation of inhomogeneous domain nucleation in epitaxial $Pb(Zr,Ti)O_3$ capacitors. *Appl. Phys. Lett.* **91,** 132903 (2007).

5. Gerra, G., Tagantsev, A. K. & Setter, N. Surface-stimulated nucleation of reverse domains in ferroelectrics. *Phys. Rev. Lett.* **94,** 107602 (2005).

6. Jesse, S. *et al.* Direct imaging of the spatial and energy distribution of nucleation centres in ferroelectric materials. *Nat. Mater.* **7,** 209–215 (2008).

7. Jo, J. Y. *et al.* Nonlinear dynamics of domain-wall propagation in epitaxial ferroelectric thin films. *Phys. Rev. Lett.* **102,** 45701 (2009).

8. Tybell, T., Paruch, P., Giamarchi, T. & Triscone, J. M. Domain wall creep in epitaxial ferroelectric $Pb(Zr_{0.2}Ti_{0.8})O_3$ thin films. *Phys. Rev. Lett.* **89,** 97601 (2002).

9. Yang, S. M. *et al.* Domain wall motion in epitaxial $Pb(Zr,Ti)O_3$ capacitors investigated by modified piezoresponse force microscopy. *Appl. Phys. Lett.* **92,** 252901 (2008).

10. Hong, S. *et al.* High resolution study of domain nucleation and growth during polarization switching in $Pb(Zr,Ti)O_3$ ferroelectric thin film capacitors. *J. Appl. Phys.* **86,** 607–613 (1999).

11. Paruch, P., Giamarchi, T. & Triscone, J. M. Domain wall roughness in epitaxial ferroelectric $PbZr_{0.2}Ti_{0.8}O_3$ thin films. *Phys. Rev. Lett.* **94,** 197601 (2005).
